# The Future of Phenomics

**DOI:** 10.1093/af/vfaa013

**Published:** 2020-04-01

**Authors:** Christine Baes, Flavio Schenkel

**Affiliations:** 1 Department of Animal Biosciences, University of Guelph, Guelph, Canada; 2 Institute of Genetics, Vetsuisse Faculty, University of Bern, Bern, Switzerland

Advances in genomics have led to an improved understanding of genetic variation in livestock production traits. In this context, collection of high-throughput, accurate phenotypic data has become the limiting factor in livestock genomics and related fields. To improve understanding of the complex interactions and underlying biological and physiological systems within livestock species, improved trait definitions of specific, economically relevant phenotypes are required. Collecting both high-density phenotypic and environmental data is therefore a major challenge for livestock production research. Novel phenotypes of interest, from gene expression to animal product characteristics, need to be identified, standardized, and their collection automated in computable formats. Development of high-throughput data collection techniques from multiple research disciplines at different biological levels is required. Research networks between academia, government, and the private sector should enhance scientific collaboration and catalyze development of modern data sharing policies. New bioinformatics approaches and advanced data management, processing, and analysis methods have become essential for integrating and interpreting the large amounts of data generated by multiple sources. Such unprecedented advances should allow a better understanding of the phenome, as well as advances to economically important traits for livestock production systems.

Included in this issue of *Animal Frontiers* are seven review articles showcasing how phenomics will impact livestock production in the future (Figure 1). The contributions from Africa, Europe, Asia, and the Americas provide a global perspective of how livestock scientists view the automation of phenotype recording.

**Figure 1.**
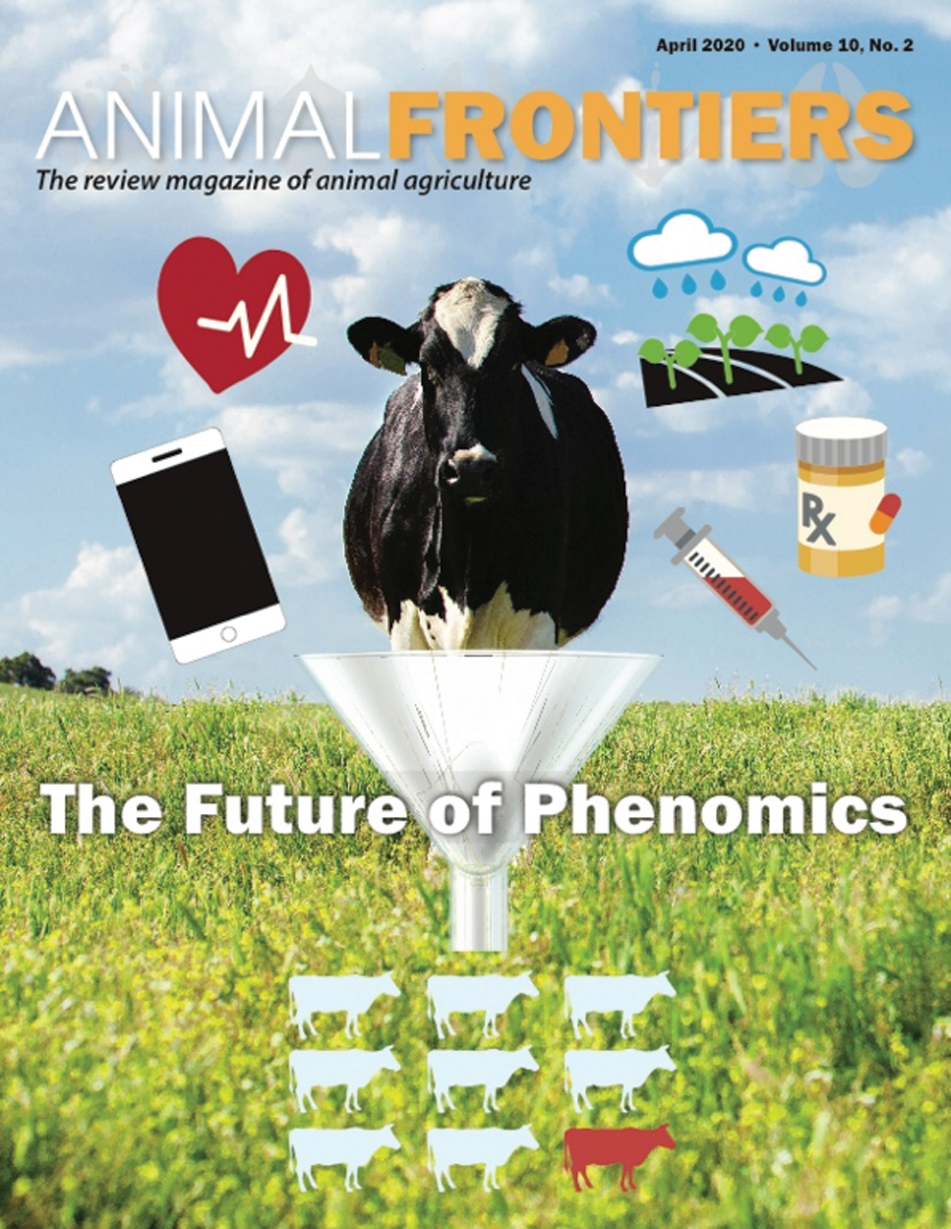
The future of phenomics will include development of high-throughput data collection techniques from multiple research disciplines at different biological levels, collection of environmental data, and new computational approaches to integrate and interpret large amounts of data.

The first two reviews offer contributions from Kenya and South Africa. Dr Raphael Mrode from the International Livestock Research Institute (Kenya) and Scotland’s Rural College (United Kingdom) and his colleagues provide excellent insight into how digital technology could change livestock development in low-income countries by examining innovative applications of emerging trends (Mrode et al., 2020). Dr Carina Visser and her colleagues from the University of Pretoria describe phenomics for sustainable production in the South African beef and dairy cattle industry (Visser et al., 2020). We then move to Europe, where Mike Coffey from Scotland’s Rural College (United Kingdom) coined the phrase “in the age of the genotype, #PhenotypeIsKing”, a hashtag that has been widely spread throughout the genetics and genomics world (Coffey, 2020). Dr Anita Seidel and her colleagues from the Christian Albrecht University in Kiel, Germany provide insight into dealing with complexity in modern dairy cattle breeding (Seidel et al., 2020). Dr Yachun Wang and her colleagues from China Agricultural University describe future opportunities and their implications for genetic improvement of temperament traits in dairy cattle (Chang et al., 2020). From there, Dr John Cole of the United States Department of Agriculture and collaborators describe the future of phenomics in the American dairy cattle industry (Cole et al., 2020). The issue is completed with Dr Ricardo Ventura and his team’s description of the opportunities and challenges of phenomics applied to livestock and aquaculture breeding in South America (Ventura et al., 2020).

The overall goal of this issue of *Animal Frontiers* is to provide insight into emerging trends in livestock phenomics and to offer viewpoints from some of the leading researchers in the field on how to use phenomics in livestock agriculture. It is clear that the pressure to improve animal housing and breeding strategies will only increase in the future, so the need to critically evaluate new strategies at the farm level is imperative. The initial research findings showcased in this issue are exciting and suggest that the future of data collection using new approaches and technologies is a bright one. Precision phenomics will not come from one technology, but an integrated approach involving many different levels of farm management, public policy, and industry commitment. Are you ready for the future?

## About the Authors

**Christine Baes** is an associate professor at the University of Guelph, NSERC Canada Research Chair in Livestock Genomics, and 2020 President of the Canadian Society of Animal Science (CSAS). She completed her PhD at the Christian Albrechts University in Kiel, Germany and worked for a number of breeding and genetics companies in Germany and Switzerland prior to returning to academia at the University of Guelph. Her current research focuses on the development of breeding programs to improve the health, welfare, and productivity of dairy cattle and poultry. **Corresponding author:**cbaes@uoguelph.ca

****

**Flavio Schenkel** is Professor at the University of Guelph, where he serves as the Director of the Centre for Genetic Improvement of Livestock, and is 2020 President Elect of the Canadian Society of Animal Science. He serves on a number of influential academic and industry boards in Canada. His research interests range from theoretical to applied genetics and genomics in livestock breeding, with a current focus on the use of genomic information to enhance genetic evaluation of livestock species with emphasis on genomic selection.

****
